# Por Que Tratar Formas Crônicas da Doença de Chagas com Benzonidazol se as Reações Adversas são Muito Frequentes?

**DOI:** 10.36660/abc.20240426

**Published:** 2024-09-06

**Authors:** Alejandro Marcel Hasslocher-Moreno

**Affiliations:** 1 Fundação Oswaldo Cruz Instituto Nacional de Infectologia Evandro Chagas Rio de Janeiro RJ Brasil Fundação Oswaldo Cruz – Instituto Nacional de Infectologia Evandro Chagas, Rio de Janeiro, RJ – Brasil

**Keywords:** Doença de Chagas, Terapêutica, Toxidermias

Após o controle bem sucedido da transmissão vetorial e transfusional da doença de Chagas (DC) no Brasil e em outros países endêmicos da América Latina, os desafios atuais são o controle da transmissão congênita da doença.^1^ Porém, ainda temos um grande contingente de pessoas infectadas com o *Trypanosoma cruzi* na forma crônica, estimados somente no Brasil em ~3,7 milhões de indivíduos, sendo ~590 mil mulheres em idade fértil.^2^

Dois guias de tratamento para DC foram produzidos pela Organização Panamericana de Saúde e pelo Ministério da Saúde do Brasil em 2018.^3,4^ Estes guias definem graus de recomendação para o tratamento tripanocida, com ênfase em recém natos, crianças e adolescentes, mulheres em idade fértil e adultos na forma clínica indeterminada até 50 anos de idade. Estudos nessas populações comprovam a eficácia do tratamento etiológico na forma crônica da DC que conduz à cura em crianças, impacta na transmissão vertical e diminui o risco de progressão da doença para a forma cardíaca.^5-7^

Para o tratamento etiológico da DC somente duas drogas estão disponíveis: nifurtimox (NTX) e benzonidazol (BZN), ambas produzidas no final da década de 1960.^8^ No Brasil, o BZN é produzido pelo Lafepe, vinculado à Secretaria Estadual de Saúde de Pernambuco, e é a droga de primeira escolha no tratamento da DC, Este medicamento, frequentemente, apresenta reações adversas medicamentosas (RAM), determinando a interrupção do tratamento em torno de 20% dos pacientes.^9^

Para adequada abordagem das RAMs é necessário utilizar um algoritmo que avalie a causalidade e outro a intensidade da reação.^10,11^ Sintomas inespecíficos como cefaleia, náuseas e dor abdominal podem estar frequentemente presentes, porém a manifestação clínica muito frequente (≥ 10%) decorrente da RAM ao BZN é a dermatite, responsável pela maioria das suspenções de tratamento. Outra manifestação frequente (≥ 1% e < 10%) é a neuropatia periférica.^9^ As manifestações inespecíficas tendem a surgir nos primeiros dias, a dermatite a partir da segunda semana, enquanto a neuropatia a partir do segundo mês de tratamento.

Em função da alta frequência de RAMs observadas com o uso do BZN e da relevância de tratar indivíduos na forma crônica da DC, é fundamental o manejo clínico das RAMs ao longo do tratamento, garantindo a segurança do paciente e permitindo que este complete os 60 dias propostos nos guias terapêuticos.^3,4^ Para tal, um protocolo clínico de seguimento deve ser considerado ao longo do tratamento, incluindo uma avaliação laboratorial antes, durante e no final do tratamento, assim como a identificação precoce da RAM e seu manejo clínico adequado.^12^

Outro aspecto importante para o sucesso do tratamento é a relação médico paciente que deve ser estabelecida antes de iniciar o processo terapêutico com BZN. Inicialmente, o paciente deve ser esclarecido da importância de seu tratamento, explicando os benefícios potenciais a curto (crianças), médio (mulheres em idade fértil) e longo (pacientes na forma indeterminada) prazos. Um segundo ponto é alertar o paciente da possibilidade deste apresentar RAMs ao BZN, em especial a dermatite, de forma que este esteja atento e informe qualquer sintoma novo ao serviço de saúde que o acompanha. Terceiro, reforçar que o relato da RAM deve ser feito o mais breve possível e que nesta condição o paciente será avaliado pelo seu médico assistente que poderá receitar sintomáticos ou até suspender o tratamento temporariamente ou definitivamente. Importante salientar que mesmo existindo um protocolo de manejo clínico, “cada caso é um caso” e, portanto, a conduta médica deve ser individualizada para cada paciente.

Novas abordagens já estão sendo desenvolvidas para a questão das RAMs no tratamento da DC. Uma delas é a aplicação de um escore de risco para RAM no uso de BZN.^13^ Este escore sinaliza que mulheres, de baixa escolaridade, brancas, entre 20 e 40 anos têm um risco maior de apresentar RAMs. Contudo este escore carece de validação externa. Outra abordagem envolve aspectos farmacogenéticos, considerando que reações de hipersensibilidade tardia estão associadas a um antígeno (HLA) classe I. No caso específico de RAMs moderadas a graves no uso de BZN, parece haver uma associação com a presença do alelo HLA-B*35 em pacientes que expressam esse polimorfismo.^8^ Além disso, estudo de farmacocinética podem auxiliar a compreender questões de toxicidade do BZN que poderia justificar, em parte, as RAMs.^14^ Por último, o uso de medicamentos reposicionados ou combinação de medicamentos pode fornecer um tratamento tripanocida com menos RAMs, mantendo a sua eficácia.^15^
