# Supplementary material for: Por Que Tratar Formas Crônicas da Doença de Chagas com Benzonidazol se as Reações Adversas são Muito Frequentes?
Source: Arq Bras Cardiol. 2024 Sep 6;121(8):e20240426. [Article in Portuguese] doi: 10.36660/abc.20240426 (PMC11495818; doi:10.36660/abc.20240426)
Supplement: Translation

# Translation: Why Treat Chronic Forms of Chagas Disease with Benznidazole if Adverse Reactions are Very Frequent?

**DOI:** 10.36660/abc.20240426i

**Authors:** Alejandro Marcel Hasslocher-Moreno

**Affiliations:** 1 Brazil Fundação Oswaldo Cruz – Instituto Nacional de Infectologia Evandro Chagas, Rio de Janeiro, RJ – Brazil

**Keywords:** Chagas Disease, Therapeutics, Drug Eruptions

After the successful control of vector and transfusion transmission of Chagas disease (CD) in Brazil and other endemic countries in Latin America, the current challenge is the control of congenital transmission of the disease.^1^ However, we still have a large contingent of people infected with *Trypanosoma cruzi* in the chronic form, estimated at around 3.7 million individuals in Brazil alone, with approximately 590,000 of them being women of childbearing age.^2^

Two treatment guidelines for CD were produced by the Pan American Health Organization and the Brazilian Ministry of Health in 2018.^3,4^ These guidelines define the degrees of recommendation for trypanocidal treatment, with an emphasis on newborns, children and adolescents, women of childbearing age, and adults with the indeterminate clinical form up to 50 years of age. Studies in these populations have proven the efficacy of etiological treatment in the chronic form of CD, which leads to a cure in children, impacts vertical transmission, and reduces the risk of progression of the disease to the cardiac form.^5-7^

For the etiological treatment of CD, only two drugs are available: nifurtimox (NFX) and benznidazole (BZN), both produced in the late 1960s.^8^ In Brazil, BZN is produced by Lafepe, linked to the Pernambuco State Department of Health, and is the first-choice drug for the treatment of CD. This medication frequently causes adverse drug reactions (ADRs), leading to the discontinuation of treatment in around 20% of patients.^9^

For an adequate approach to ADRs, it is necessary to use an algorithm that assesses causality and another that assesses the intensity of the reaction.^10,11^ Non-specific symptoms such as headache, nausea, and abdominal pain are frequently present; however, the very frequent clinical manifestation (≥ 10%) resulting from ADR to BZN is dermatitis, which is responsible for most treatment suspensions. Another frequent manifestation (≥1% and <10%) is peripheral neuropathy.^9^ Non-specific manifestations tend to appear in the first few days, dermatitis from the second week onwards, while neuropathy appears from the second month of treatment.

Due to the high frequency of ADRs observed with the use of BZN and the importance of treating individuals in the chronic form of CD, it is essential to manage ADRs clinically throughout the treatment, ensuring patient safety and allowing them to complete the 60 days proposed in the therapeutic guidelines.^3,4^ To this end, a clinical follow-up protocol should be considered throughout the treatment, including a laboratory evaluation before, during, and at the end of the treatment, as well as the early identification of ADRs and their appropriate clinical management.^12^

Another important aspect of the success of treatment is the doctor-patient relationship that should be established before starting the therapeutic process with BZN. Initially, the patient must be informed about the importance of their treatment, explaining the potential short-term (children), medium-term (women of childbearing age), and long-term (patients with the indeterminate form) benefits. A second point is to alert the patient to the possibility of experiencing ADRs with BZN, especially dermatitis, so they are vigilant and report any new symptoms to the healthcare provider accompanying them. Third, emphasize that reporting ADRs should be done as soon as possible, and in this situation, the patient will be evaluated by their attending physician who may prescribe symptomatic treatments or even temporarily or permanently suspend treatment. It is important to note that even with a clinical management protocol in place, “each case is unique”, and therefore, medical management should be tailored to each patient.

New approaches are already being developed for addressing ADRs in the treatment of CD. One of them is the application of a risk score for ADRs in the use of BZN.^13^ This score indicates that women of low educational attainment, Caucasian ethnicity, aged between 20 and 40 years, have a higher risk of experiencing ADRs. However, this score lacks external validation. Another approach involves pharmacogenetic aspects, considering that delayed hypersensitivity reactions are associated with a class I antigen (HLA). In the specific case of moderate to severe ADRs in BZN use, there appears to be an association with the presence of the HLA-B*35 allele in patients expressing this polymorphism.^8^ Additionally, pharmacokinetic studies can help understand issues of BZN toxicity, which could partly explain ADRs.^14^ Finally, the use of repurposed drugs or drug combinations may provide trypanocidal treatment with fewer ADRs while maintaining efficacy. ^15^
